# BSA-seq-based identification of candidates associated with stable resistance to *Ralstonia pseudosolanacearum* in pepper

**DOI:** 10.3389/fpls.2026.1956689

**Published:** 2026-09-29

**Authors:** Ji-Su Kwon, Chamini Wijethunga, Junesung Lee, Yujin Sim, Myung-Shin Kim, Seon-In Yeom, Won-Hee Kang

**Affiliations:** 1 Department of Horticulture, Division of Applied Life Science (BK21 Four), Institute of Agriculture & Life Science, Gyeongsang National University, Jinju, Republic of Korea; 2 Department of Biosciences and Bioinformatics, Myongji University, Yongin, Republic of Korea

**Keywords:** bacterial wilt, bulk segregant analysis (BSA), *Capsicum annuum*, quantitative trait loci, *Ralstonia pseudosolanacearum*

## Abstract

Bacterial wilt is a major constraint on pepper production, and the development of resistant cultivars is the most effective and sustainable strategy for disease management. However, pepper germplasm with stable resistance and its genetic basis remain insufficiently characterized. In this study, 63 pepper accessions were screened for bacterial wilt resistance using a leaf-to-whole plant bioassay. Based on disease severity index values, seven accessions were classified as extremely resistant (ER), 25 as resistant, 22 as moderately susceptible, and nine as susceptible. Selected accessions were further evaluated against five *Ralstonia pseudosolanacearum* strains with distinct pathogenic and genetic characteristics. Most ER accessions maintained strong resistance across diverse pathogen backgrounds, although their resistance levels varied among strains, suggesting the involvement of multiple resistance factors. To identify resistance-associated genomic regions, bulked segregant analysis sequencing (BSA-seq)-based QTL analysis was performed using an F_2_ population of 398 individuals derived from a cross between an ER accession (‘MC4’) and a susceptible accession (‘Subicho’). Two candidate regions were identified on chromosomes 8 and 11. These regions contained candidate genes encoding *LCB1-like*, *CABIN1*, *NAR1*, and *G3BP-like* proteins. These findings provide useful genetic resources and candidate loci for further investigation and breeding of bacterial wilt-resistant pepper.

## 1 Introduction

Bacterial wilt, caused by members of the *Ralstonia solanacearum* species complex (RSSC), is one of the most destructive diseases affecting solanaceous crops worldwide (Vailleau and Genin, 2023). RSSC strains exhibit substantial pathogenic and genetic diversity, and *Ralstonia pseudosolanacearum* includes strains formerly classified as phylotypes I and III (Safni et al., 2014; Sharma et al., 2022). The pathogen colonizes vascular tissues and causes rapid wilting and plant death (Balamurugan et al., 2021). Because of its broad host range, persistence in soil, and environmental adaptability, bacterial wilt is difficult to control using conventional management practices (Ahmed et al., 2022). Therefore, the development of resistant cultivars remains the most effective and sustainable strategy for disease management (Abu Bakar et al., 2025).

Pepper (*Capsicum* spp.) is an economically important vegetable crop cultivated worldwide (Liu et al., 2013; Tripodi and Kumar, 2019). However, bacterial wilt can cause substantial yield losses, particularly under warm and humid conditions (Thakur et al., 2021; Kapoor et al., 2023). Previous studies have identified pepper germplasm with resistance to bacterial wilt, including accessions exhibiting biovar-specific or broad-spectrum resistance (Lebeau et al., 2011; Ro et al., 2024). Nevertheless, resistance responses can vary depending on the pathogen strain. *R. solanacearum* isolates have been classified into distinct groups based on their biovar and pathogenicity, and pepper cultivars frequently show differential responses to individual isolates (Lee et al., 2018; Naveena et al., 2020). These findings indicate that resistance evaluation using multiple strains with distinct pathogenic and genetic backgrounds is important for identifying stable resistance sources for breeding (Chae et al., 2022; Lee et al., 2024).

Bacterial wilt resistance in pepper is generally controlled by multiple genetic factors. Previous studies have identified resistance-associated quantitative trait loci (QTLs) using different resistant accessions, segregating populations, and pathogen isolates (Du et al., 2019; Chae et al., 2022; Lee et al., 2022, 2024). Importantly, the detected QTLs can differ depending on the resistance donor and pathogen strain, suggesting that multiple resistance factors contribute to bacterial wilt resistance in pepper (Lavale et al., 2022). The availability of diverse germplasm resources and high-throughput sequencing technologies provides new opportunities to discover resistance-associated loci and candidate genes with greater resolution (Varshney et al., 2021; Kwon et al., 2024). Bulked segregant analysis sequencing (BSA-seq), which compares allele frequencies between resistant and susceptible bulks derived from a segregating population, provides an efficient approach for identifying genomic regions associated with complex traits (Takagi et al., 2013; Zhang et al., 2023). This approach is particularly useful for prioritizing candidate genomic regions and genes associated with bacterial wilt resistance.

In this study, we first screened 63 pepper accessions for bacterial wilt resistance using a leaf-to-whole plant bioassay, an established method for evaluating pepper responses to *Ralstonia* infection, following inoculation with *R. pseudosolanacearum* strain SL1931. Selected extremely resistant accessions were subsequently evaluated against five *R. pseudosolanacearum* strains with distinct pathogenic and genetic characteristics to assess the stability of resistance across diverse pathogen backgrounds. Finally, BSA-seq-based QTL analysis was performed using an F_2_ population derived from a cross between a strongly resistant accession and a susceptible accession. This study identifies stable resistance sources and provides candidate genomic regions and genes for further investigation of bacterial wilt resistance and resistance breeding in pepper.

## 2 Materials and methods

### 2.1 Plant materials and growth conditions

A total of 63 pepper accessions, including breeding lines, landraces, wild accessions, and commercial cultivars (**Supplementary Table 1**), were evaluated in this study. These accessions were obtained from the Rural Development Administration (RDA, Republic of Korea), the World Vegetable Center (formerly AVRDC), and the Molecular Breeding Laboratory (MBLAB) at Gyeongsang National University, Republic of Korea. Three pepper cultivars were used as controls including the bacterial wilt-resistant cultivar *Capsicum annuum* ‘MC4’ and the susceptible cultivars ‘Subicho’ and ‘Geonchowang’. Plants were grown for 3–4 weeks in a controlled growth chamber maintained at 30 ± 1 °C under a 16 h light/8 h dark photoperiod with 50% relative humidity.

### 2.2 Bacterial inoculum preparation

For the initial screening, *R. pseudosolanacearum* strain SL1931 (KACC10711; phylotype I, biovar 3) was obtained from Dr. Seon-Woo Lee (Dong-A University, Republic of Korea). Bacterial cells were streaked onto Kelman’s tetrazolium chloride (TZC) agar medium and incubated at 28 °C for 48 h. A single fluidal colony was subsequently cultured in CPG broth at 28 °C with shaking at 250 rpm for 24 h. The bacterial suspension was initially adjusted to an OD_600_ of 0.3 (corresponding to ~3 × 10^8^ CFU mL^-1^) and subsequently diluted with sterile distilled water to achieve a final working concentration of 10^6^ CFU mL^-1^. To assess the stability of resistance to diverse bacterial wilt pathogens, selected pepper accessions were inoculated with five phylotype I *R. pseudosolanacearum* strains representing biovars 3 and 4 (SL1931, HWA, AD, SL3505, and SL341), all which were isolated in Korea (She et al., 2017). Strain SL341 was obtained from Dr. Seon-Woo Lee (Dong-A University, Republic of Korea) (Cho et al., 2018). Strains HWA, AD, and SL3505 were provided by Dr. Jun-Dae Lee and have been used in previous studies investigating bacterial wilt resistance and QTL identification in pepper (Lee et al., 2018).

### 2.3 Leaf-to-whole plant bioassay

A leaf-to-whole plant bioassay (LWB) was used to assess bacterial wilt resistance (Kwon et al., 2021). Pepper plants at the four-leaf stage were infiltrated with bacterial suspensions at a concentration of 10^6^ CFU mL^−1^. The third and fourth fully expanded leaves were infiltrated on the abaxial surface with bacterial suspension using a 1 mL needleless syringe over a similar leaf area, corresponding to approximately 0.1 mL per leaf. Following inoculation, plants were maintained under controlled conditions (30 ± 1 °C, 50% relative humidity, and a 16 h light/8 h dark photoperiod) for disease evaluation. Disease development was monitored for 20 days after inoculation (DAI). Three independent biological experiments were conducted, and each experiment included 8–16 plants per accession.

### 2.4 Disease evaluation and statistical analysis

Disease severity was assessed daily for 20 DAI using a disease severity index (DSI) ranging from 0 to 4. A score of 0 indicated no visible symptoms; 1 indicated wilting of one or two inoculated leaves; 2 indicated wilting of three or four leaves; 3 indicated wilting of five or six leaves; and 4 indicated severe wilting, stem collapse, or plant death. Mean DSI values, wilt rate (WR), and area under the disease progress curve (AUDPC) were calculated to evaluate disease progression. Accessions were classified based on mean DSI values as follows: extreme resistant (ER), DSI ≤ 1; resistant (R), 1 < DSI ≤ 2; moderately susceptible (MS), 2 < DSI ≤ 3; and susceptible (S), DSI > 3.

For resistance evaluation against the five *R. pseudosolanacearum* strains, phenotypic classification was based on WR. Accessions with WR ≤ 10% were classified as extreme resistant (ER), 10% < WR ≤ 30% as resistant (R), 30% < WR ≤ 50% as moderately resistant (MR), 50% < WR ≤ 70% as moderately susceptible (MS), and WR > 70% as susceptible (S). Data were subjected to one-way analysis of variance (ANOVA), followed by Duncan’s multiple range test at *P* < 0.05. Statistical analyses were performed using Python and associated statistical libraries.

### 2.5 BSA-seq and SNP-index analysis

BSA-seq-based QTL analysis was performed using whole-genome resequencing of an F_2_ population (n = 398) derived from a cross between the susceptible cultivar ‘Subicho’ and the resistant cultivar ‘MC4’. All F_2_ individuals were evaluated for bacterial wilt resistance using the leaf-to-whole plant bioassay. Individuals from the extreme ends of phenotypic distribution were selected for bulk construction. Two DNA bulks were constructed by pooling equal amounts of genomic DNA from 30 highly resistant individuals (DSI = 0–1 at 20 DAI; R-pool) and 30 highly susceptible individuals exhibiting severe wilting symptoms (DSI = 4 at 20 DAI; S-pool).

Genomic DNA from the parental lines and the two bulks was extracted using the CTAB method. Sequencing libraries were prepared by fragmenting genomic DNA, followed by end repair, A-tailing, adapter ligation, and size selection. Libraries were sequenced using a 150-bp paired-end strategy on the Illumina HiSeq X Ten platform (Seeders Co., Daejeon, Republic of Korea). Raw sequencing reads were processed using trimmomatic (v0.40) in QTL-seq (v2.2.2) (Takagi et al., 2013; Bolger et al., 2014). Quality filtering was performed using the following parameters: minimum quality score (-q) of 30 and minimum percentage of bases meeting the quality threshold (-p) of 90. Only high-quality reads were retained for downstream analyses.

To generate a customized reference sequence, high-quality reads from the susceptible parent were aligned to the pepper reference genome using BWA (v0.7.17) (Jung and Han, 2022), and resulting alignment files were processed using SAMtools (v1.7-1) (Li et al., 2009). SNPs were identified and filtered using bcftools (v1.8) (Danecek et al., 2021). Validated polymorphisms were subsequently incorporated into the reference sequence to construct a customized susceptible-parent reference genome. Unless otherwise stated, all analyses were performed using default parameters.

The SNP-index for each SNP position was calculated for both resistant and susceptible bulks according to the QTL-seq pipeline. A sliding window analysis was performed using a window size of 300 kb with a 10 kb increment. SNPs with read depths between 15 and 100 were retained for analysis. The ΔSNP-index (R - S) was calculated by subtracting the SNP-index of the susceptible bulk from that of the resistant bulk. Statistical confidence intervals were estimated using 5,000 permutation tests, and genomic regions exceeding the 99% confidence interval were considered candidate QTL regions associated with bacterial wilt resistance. Genes located within significant QTL regions were examined for sequence polymorphisms between the parental lines. QTL regions exhibiting negative ΔSNP-index values, indicating enrichment of resistant-parent alleles, were selected for further analysis. SNP-index and ΔSNP-index plots were generated using qtlplot and custom R scripts.

## 3 Results

### 3.1 Screening of pepper germplasm identifies seven extremely resistant accessions

A total of 63 pepper accessions were evaluated for bacterial wilt resistance using the leaf-to-whole plant bioassay following inoculation with *Ralstonia pseudosolanacearum* strain SL1931 (**Figure 1**; **Supplementary Tables 1**, **2**). Disease progression was monitored for 20 days after inoculation (DAI), and disease severity was assessed using a disease severity index (DSI) ranging from 0 to 4 (**Figure 1A**; **Supplementary Table 2**). Disease responses varied substantially among the accessions. The resistant control ‘MC4’ maintained a final DSI value of 0.9, whereas the susceptible control ‘Subicho’ reached a DSI value of 4.0 at 20 DAI. Disease symptoms generally appeared between 4 and 7 DAI and progressed rapidly in susceptible accessions during the second week after inoculation.

**Figure 1:**
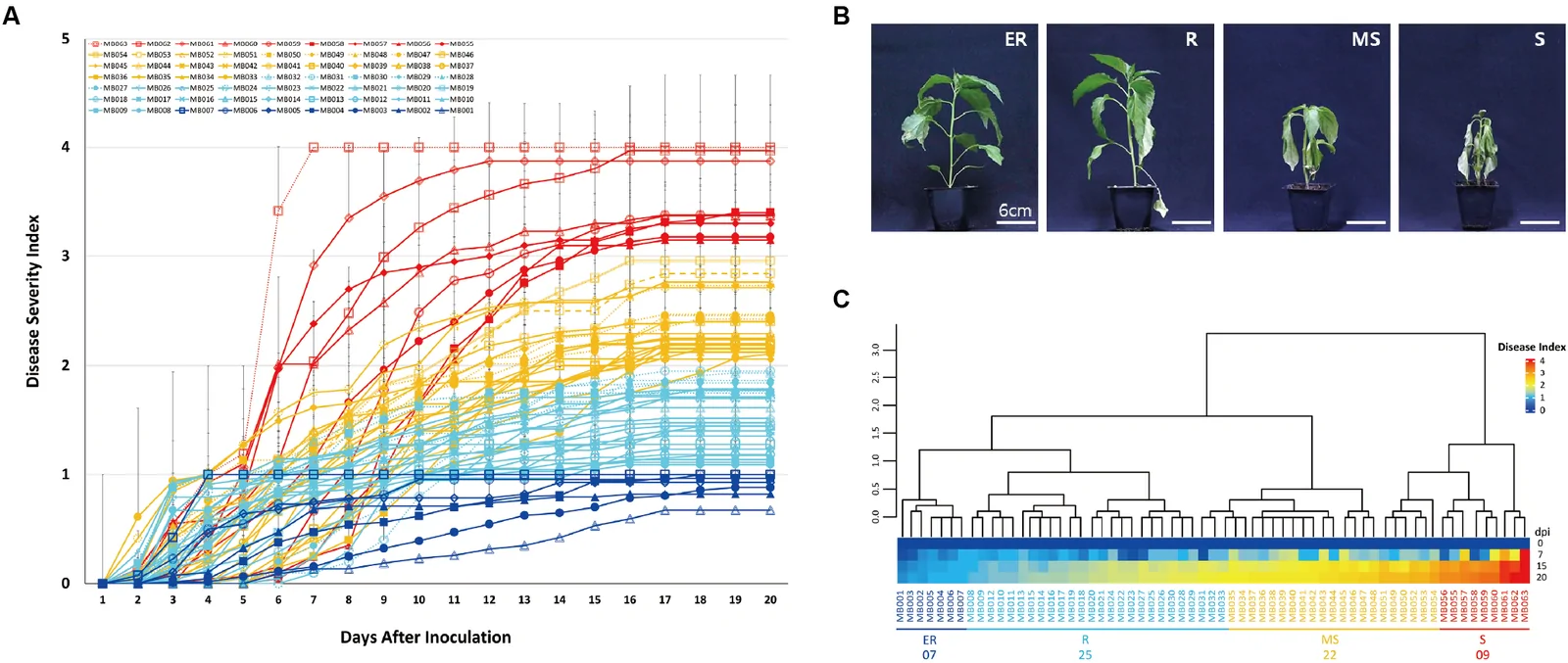
Disease progression and resistance classification of 63 pepper accessions following inoculation with *Ralstonia pseudosolanacearum* strain SL1931. **(A)** Disease progression in 63 pepper germplasm accessions following inoculation with *R. pseudosolanacearum* strain SL1931 (phylotype I), assessed using the leaf-to-whole bioassay (LWB). Each data point represents the mean disease severity index (DSI) from the three independent experiments, with 10–15 plants evaluated per experiment. Error bars indicate the standard deviation (SD) among the three independent experiment. Resistance categories are defined as follows: extreme resistant (ER; DSI ≤ 1, blue line), resistant (R; 1 < DSI ≤ 2, skyblue line), moderately susceptible (MS; 2 < DSI ≤ 3, yellow line), and susceptible (S; DSI > 3, red line). **(B)** Representative images of BW disease symptoms, categorized as ER, R, MS, and S, for phenotype evaluation. The white scale bar represents 6 cm. **(C)** Hierarchical clustering of 63 pepper accessions based on DSI scores recorded at 0, 7, 15, and 20 DAI. The heatmap was generated using DSI scores (0–4) recorded at 0, 7, 15, and 20 days after inoculation (DAI). Red indicates susceptibility, while blue represents resistance.

Based on DSI values at 20 DAI, seven accessions were classified as extreme resistant (ER), 25 as resistant (R), 22 as moderately susceptible (MS), and nine as susceptible (S) (**Figure 1A**; **Supplementary Table 2**). Representative disease symptoms corresponding to each resistance category are shown in **Figure 1B**. ER accessions exhibited little or no disease progression beyond the infiltrated leaves, whereas susceptible accessions developed extensive leaf wilting, stem collapse, and eventual plant death. Among the ER accessions, ‘K1’ (MB001) and ‘Chilly Bangi’ (MB002) exhibited the strongest resistance responses, with final DSI values of 0.7 and 0.8, respectively, which were lower than that of the resistant control ‘MC4’. In contrast, ‘PI159236’ (MB063) reached a DSI value of 4.0 as early as 7 DAI, exhibiting the most rapid disease progression among the evaluated accessions, whereas ‘Subicho’ (MB062) also developed complete wilting and plant death, resulting in a final DSI value of 4.0.

Hierarchical clustering analysis using DSI values recorded at 0, 7, 15, and 20 DAI clearly separated resistant and susceptible accessions into distinct groups (**Figure 1C**). Accessions classified as ER and R clustered together and exhibited limited disease progression, whereas MS and S accessions formed separate clusters characterized by progressively increasing disease severity. Consequently, seven accessions classified as extremely resistant (ER) were selected for further resistance stability assessment.

### 3.2 Multi-strain evaluation confirms stable resistance and reveals accession-specific variation

To evaluate the stability of resistance across diverse pathogen backgrounds, 13 selected pepper accessions were inoculated with five *R. pseudosolanacearum* strains with distinct pathogenic and genetic characteristics: SL1931, HWA, AD, SL3505, and SL341 (**Figure 2**; **Table 1**). The selected accessions included seven extreme resistant accessions, two resistant accessions, three susceptible accessions, and the susceptible control cultivar ‘Geonchowang’.

**Figure 2:**
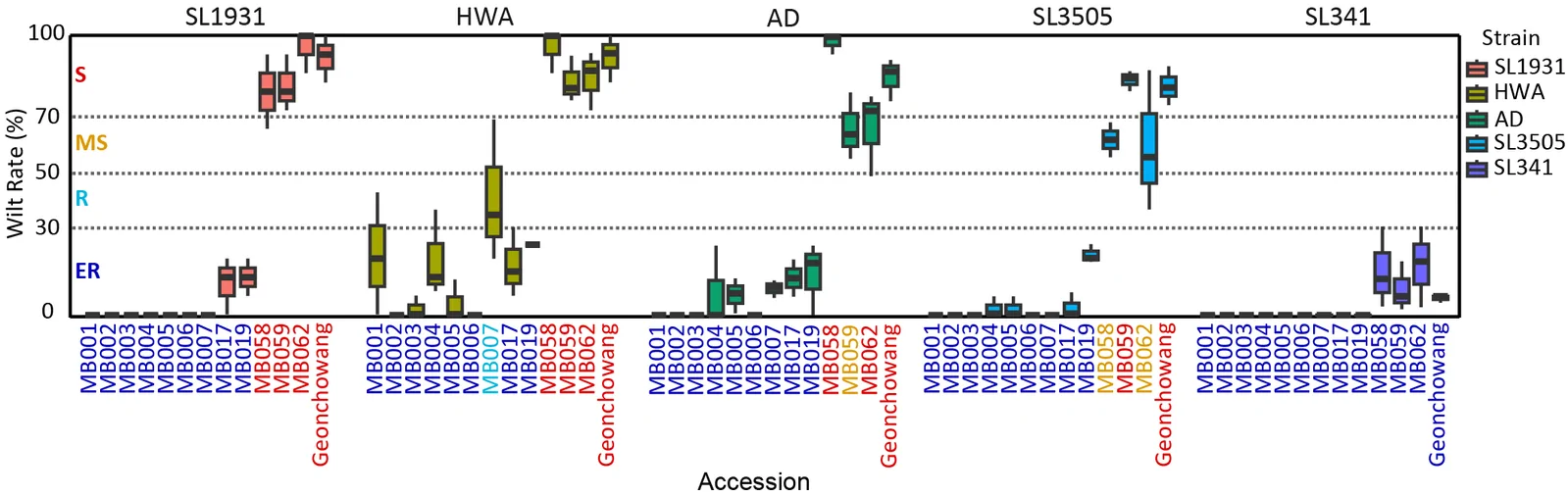
Validation of bacterial wilt resistance in 13 pepper accessions using five *Ralstonia pseudosolanacearum* strains. Thirteen pepper accessions selected from the initial screening were inoculated with five *R. pseudosolanacearum* strains at an inoculum concentration of 10^6^ CFU mL^−1^. Leaf inoculation assays were conducted in three independent experiments, with 8–16 plants per isolate in each experiment. Wilt rate (WR) was categorized as follows: extreme resistant (ER; WR ≤ 10%), resistant (R; 10% < WR ≤ 30%), moderately resistant (MR; 30% < WR ≤ 50%), moderately susceptible (MS; 50% < WR ≤ 70%), and susceptible (S; WR > 70%). Colored boxes represent the five *R. pseudosolanacearum* strains used in this study. The colors of the accession names below the box plots indicate their corresponding wilt rate categories.

**Table 1:** Wilt rates and resistance classifications of selected pepper accessions inoculated with five *Ralstonia pseudosolanacearum* strains.

Pathogenicity in pepper	Highly pathogenic		Highly pathogenic		Moderately pathogenic		Moderately pathogenic		Weakly pathogenic	
Strain (biovar)	SL1931		HWA		AD		SL3505		SL341	
Accession	(Biovar 3)		(Biovar 4)		(Biovar 3)		(Biovar 4)		(Biovar 3)	
MB001 - K1	0^a^ ± 0^b^	ER^c^	21.3 ± 21.9	R	0 ± 0	ER	0 ± 0	ER	0 ± 0	ER
MB002 - Chilly Bangi	0 ± 0	ER	0 ± 0	ER	0 ± 0	ER	0 ± 0	ER	0 ± 0	ER
MB003 - MC4	0 ± 0	ER	2.2 ± 3.8	ER	0 ± 0	ER	0 ± 0	ER	0 ± 0	ER
MB004 - VP55	0 ± 0	ER	19.7 ± 15.6	R	8.3 ± 14.4	ER	2.1 ± 3.6	ER	0 ± 0	ER
MB005 - PBC384	0 ± 0	ER	4.2 ± 7.2	ER	6.5 ± 6.3	ER	2.1 ± 3.6	ER	0 ± 0	ER
MB006 - PBC1347	0 ± 0	ER	0 ± 0	ER	0 ± 0	ER	0 ± 0	ER	0 ± 0	ER
MB007 - Jeju	0 ± 0	ER	41.9 ± 25.6	MR	9.6 ± 3.1	ER	0 ± 0	ER	0 ± 0	ER
MB017 - CM334	11.1 ± 10.2	R	17.8 ± 12.5	R	13.1 ± 6.7	R	2.6 ± 4.4	ER	0.1 ± 0.1	ER
MB019 - Perennial	13.3 ± 6.7	R	25 ± 0	R	14.6 ± 13.0	R	21.3 ± 3.3	R	0 ± 0	ER
MB058 - ECW30R	80.0 ± 13.3	S	95.6 ± 7.7	S	97.9 ± 3.6	S	62.5 ± 6.3	MS	15.5 ± 14.5	R
MB059 - Dempsey	82.2 ± 10.2	S	83.7 ± 8.2	S	67.1 ± 12.0	MS	83.9 ± 3.6	S	8.9 ± 8.9	ER
MB062 - Subicho	95.6 ± 7.7	S	84.9 ± 10.5	S	67.3 ± 15.2	MS	60.4 ± 25.3	MS	17.5 ± 14.5	R
Geonchowang (control)	92.2 ± 8.4	S	92.4 ± 8.4	S	85.4 ± 7.6	S	81.7 ± 7.0	S	5.5 ± 1.3	ER

^a^ Wilting rate (%) was calculated as [The number of wilt plants/The number of total plants] x 100.

^b^ Data are presented as the mean ± SD of three independent biological replicates.

^c^ Phenotypes were classified based on wilt rate (WR) as follows: extreme resistant (ER), WR ≤ 10%; resistant (R), 10% < WR ≤ 30%; moderately resistant (MR), 30% < WR ≤ 50%; moderately susceptible (MS), 50% < WR ≤ 70%; and susceptible (S), WR > 70%.

Overall, accessions classified as ER in the initial screening maintained high levels of resistance responses against all five strains (**Figure 2**). ‘Chilly Bangi’ (MB002), ‘MC4’ (MB003), ‘PBC384’ (MB005), and ‘PBC1347’ (MB006) consistently exhibited ER phenotypes against all strains tested, with wilt rates below 5%. Similarly, ‘K1’ (MB001), ‘VP55’ (MB004), and ‘Jeju’ (MB007) exhibited an ER response to strain SL1931, whereas they displayed an R or MR response to the HWA strain, suggesting minor variation in resistance levels while maintaining overall resistance to diverse *R. pseudosolanacearum* isolates (**Table 1**). The resistant accessions ‘CM334’ (MB017) and ‘Perennial’ (MB019) maintained resistance against all five *R. pseudosolanacearum* strains tested.

In contrast, susceptible accessions generally exhibited high wilt rates following inoculation with SL1931 and HWA but lower wilt rates following inoculation with AD and SL341. Among the tested strains, SL1931 and HWA induced the strongest disease responses, whereas SL341 induced the lowest wilt rates across most accessions, including susceptible controls. Although most ER accessions maintained strong resistance across diverse pathogen backgrounds, the magnitude of resistance varied among accession–strain combinations. These results reveal accession-specific resistance profiles and suggest that multiple resistance factors may contribute to strain-dependent bacterial wilt responses in pepper.

### 3.3 BSA-seq-based QTL analysis prioritizes candidate resistance-associated regions

Among the resistant accessions evaluated, ‘MC4’ exhibited consistently high levels of resistance against all tested *R. pseudosolanacearum* strains and was therefore selected as the resistant parent for subsequent BSA-seq-based QTL analysis. An F_2_ population (n = 398) derived from a cross between the resistant cultivar ‘MC4’ and the susceptible cultivar ‘Subicho’ was evaluated following inoculation with strain SL1931. The parental lines showed clearly contrasting responses (‘MC4’, DSI ≤ 1; ‘Subicho’, DSI = 4; **Figure 3A**). F_1_ plants derived from ‘Subicho’ × ‘MC4’ were previously reported to exhibit an intermediate disease response between the two parental lines, with a mean DSI of approximately 2.5 (Kwon et al., 2021). Among the 398 F_2_ progeny evaluated at 20 DAI, 14 plants had DSI = 0, 157 had DSI = 1, 28 had DSI = 2, 4 had DSI = 3, and 195 had DSI = 4 (**Figure 3B**). This broad segregation of disease severity in the F_2_ population is consistent with the quantitative inheritance of bacterial wilt resistance.

**Figure 3:**
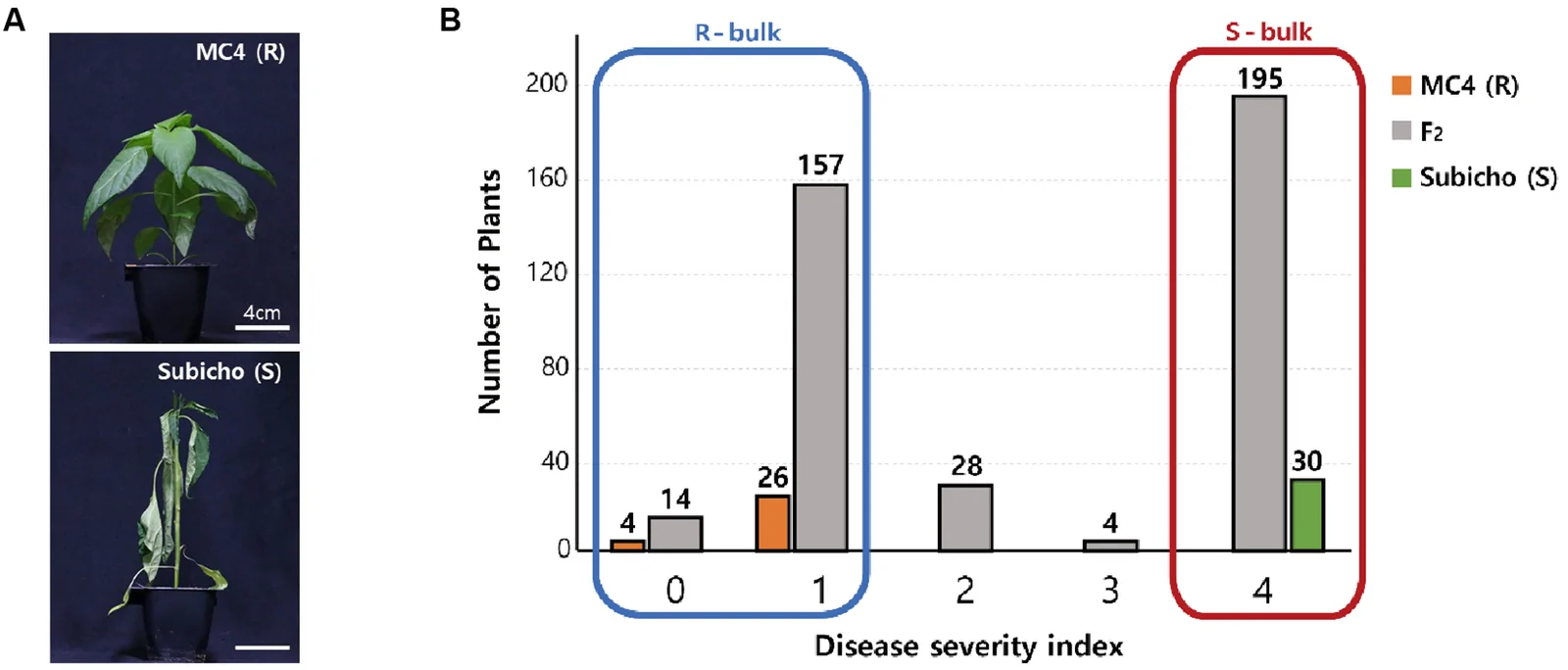
Phenotypic distribution and construction of extreme bulks in the ‘Subicho’ × ‘MC4’ F_2_ segregating population for BSA-seq-based QTL analysis. **(A)** Representative disease phenotypes of the susceptible parent ‘Subicho’ and the resistant parent ‘MC4’ at 15 days after inoculation with *R. pseudosolanacearum* strain SL1931**. (B)** Frequency distribution of bacterial wilt resistance in the ‘Subicho’ × ‘MC4’ F_2_ segregating population at 20 days after inoculation. The x-axis represents disease severity classes (DSI 0–4) and the numbers above each bar indicate the number of individuals in each disease severity class. Genomic DNA from F_2_ individuals with DSI values of 0–1 was pooled to generate the resistant bulk (R-bulk; blue box), whereas DNA from individuals with a DSI value of 4 was pooled to generate the susceptible bulk (S-bulk; red box).

Based on the phenotypic distribution, 30 highly resistant individuals with DSI values of 0–1 and 30 highly susceptible individuals with a DSI value of 4 were selected to construct resistant and susceptible bulks, respectively. Whole-genome resequencing of the two bulks generated high-quality sequencing data, with average genome coverage ranging from 39.26× to 39.48× and Q30 values exceeding 92% (**Supplementary Table 3**). After quality filtering, 24,984 single-nucleotide polymorphisms (SNPs) and 3,503 insertions and deletions (InDels) were retained for allele-frequency analysis. Statistical analysis revealed 182 significant sliding-window regions (*P* < 0.01) (**Supplementary Tables 4**, **5**). Among these significant windows, regions showing negative ΔSNP-index values consistent with enrichment of resistant-parent alleles in the resistant bulk were further prioritized.

Sliding-window analysis prioritized two candidate resistance-associated regions with negative ΔSNP-index (R − S) values (**Figure 4**; **Supplementary Tables 4**, **5**). Negative ΔSNP-index values indicate that susceptible-parent alleles are enriched in the susceptible bulk, whereas resistant-parent alleles are enriched in the resistant bulk, consistent with resistance-associated loci. The first region was located on chromosome 8 at approximately 44.2–44.8 Mb and contained genes annotated as *LCB1-like*, *CABIN1*, and *NAR1*. The second region was located on chromosome 11 at approximately 16.2–16.8 Mb and contained a *G3BP-like* gene and a gene encoding a hypothetical protein. These regions were prioritized as candidate loci for further investigation of bacterial wilt resistance in pepper.

**Figure 4:**
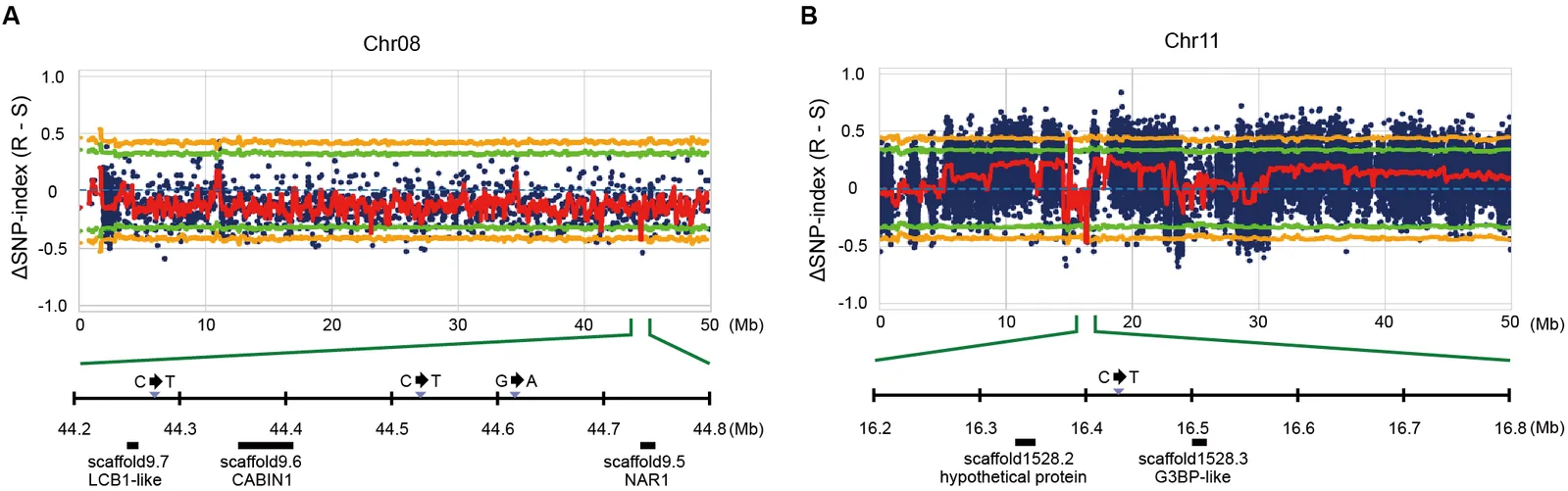
BSA-seq-based QTL analysis identifies candidate resistance-associated regions on chromosomes 8 and 11. ΔSNP-index plots for chromosome 8 **(A)** and chromosome 11 **(B)** are shown. The ΔSNP-index (R - S) was calculated using a 300 kb sliding window with a 10 kb step size (red line). Statistical confidence intervals under the null hypothesis of no QTL are indicated by the orange (*P* < 0.01) and green (*P* < 0.05) lines. Candidate genes and SNPs identified within the QTL intervals are displayed below each ΔSNP-index plot. Black arrows indicate SNPs identified in the resistant parent, and sky-blue triangles indicate the genomic positions of the corresponding variants. Black bars represent annotated gene models, together with gene names and predicted functional descriptions.

## 4 Discussion

The identification of stable resistance sources is essential for the development of bacterial wilt-resistant pepper cultivars. In this study, seven accessions were classified as extremely resistant following inoculation with *R. pseudosolanacearum*. Notably, ‘K1’ and ‘Chilly Bangi’ exhibited lower disease severity than the resistant control cultivar ‘MC4’. Because ‘MC4’ has been widely used as a resistance donor in pepper breeding and genetic studies, these accessions may represent valuable alternative sources of bacterial wilt resistance. The identification of multiple extremely resistant accessions is important because it expands the available resistance pool beyond a single donor and provides additional genetic resources for resistance breeding.

Resistance identified against a single strain does not necessarily indicate stable resistance across pathogen backgrounds (Lebeau et al., 2011). Therefore, selected accessions were further evaluated against five *R. pseudosolanacearum* strains with distinct pathogenic and genetic characteristics. Most extremely resistant accessions maintained strong resistance across these diverse pathogen backgrounds, supporting their potential value as stable resistance sources. In particular, several ER accessions showed consistently low wilt rates across the tested strains, whereas susceptible accessions displayed high disease severity against highly pathogenic strains such as SL1931 and HWA. These results indicate that the selected ER accessions are not merely resistant to a single screening strain but retain resistance across multiple pathogen backgrounds.

Although most ER accessions maintained strong resistance, their resistance levels differed depending on the pathogen strain. This accession-by-strain variation is biologically meaningful because it suggests that bacterial wilt resistance in pepper may involve multiple resistance factors rather than a single uniform mechanism. Previous studies have shown that bacterial wilt resistance in pepper is quantitatively inherited and can be influenced by the pathogen isolate used for evaluation (Du et al., 2019; Lee et al., 2022). Thus, the strain-dependent differences observed among ER accessions in this study are consistent with an oligogenic or polygenic resistance model and support the need to combine stable phenotypic screening with genetic analysis (Lafortune et al., 2005; Kwon et al., 2024).

In this study, resistance was evaluated using a leaf-to-whole plant bioassay. While natural *Ralstonia* infection occurs primarily through the roots in soil, leaf infiltration provides a complementary approach for evaluating disease responses under relatively uniform inoculation conditions. Previous studies using conventional root-drenching assays have shown that ‘MC4’ exhibits strong resistance, whereas ‘Subicho’ is highly susceptible (Hwang et al., 2017; Kwon et al., 2021). Together with the results of the present study, these findings suggest that the contrasting disease responses of ‘MC4’ and ‘Subicho’ are reproducible across different inoculation methods.

Using these contrasting parental lines, the ‘Subicho’ × ‘MC4’ F_2_ population exhibited continuous variation in disease response, supporting the quantitative nature of bacterial wilt resistance in pepper. To identify genomic regions associated with this resistance, we performed BSA-seq- based QTL analysis using extreme resistant and susceptible bulks derived from the F_2_ population. Two candidate resistance-associated regions were identified on chromosomes 8 and 11. The chromosome 8 region is particularly notable because resistance-associated loci on chromosome 8 have been reported in independent pepper mapping studies (Chae et al., 2022; Kwon et al., 2024; Lee et al., 2024). However, direct equivalence between the region identified here and previously reported loci should be interpreted cautiously because resistance donors, pathogen strains, population structures, and reference genome coordinates differ among studies (Lebeau et al., 2011). The chromosome 11 region was not consistently detected in previous studies and may represent an additional resistance-associated region in the ‘MC4’ genetic background.

Several genes located within the candidate regions have annotations potentially related to plant defense responses. The chromosome 8 region contained genes annotated as *LCB1-like*, *CABIN1*, and *NAR1*, whereas the chromosome 11 region contained a *G3BP-like* gene and a hypothetical protein. *LCB1-like* proteins are involved in sphingolipid biosynthesis, a pathway associated with defense signaling and programmed cell death during plant–pathogen interactions (Saucedo‐García et al., 2011; Berkey et al., 2012). *G3BP-like* proteins have also been implicated in plant immunity and stress-associated RNA regulation (Abulfaraj et al., 2018). Although these annotations provide biological hypotheses, the presence of these genes within BSA-seq candidate regions does not establish causal relationships with bacterial wilt resistance. Therefore, these genes should be regarded as prioritized candidates for subsequent genetic, expression, and functional analyses.

Collectively, this study provides two complementary outputs for bacterial wilt resistance breeding in pepper. First, it identifies pepper germplasm with stable resistance across diverse pathogen backgrounds. Second, it prioritizes candidate resistance-associated genomic regions and genes using BSA-seq-based QTL analysis. These findings provide useful resources for further investigation of bacterial wilt resistance and for the development of resistant pepper cultivars.

## Data Availability

The datasets generated for this study can be found in the NCBI Sequence Read Archive under BioProject accession number PRJNA1503100 and are available at [https://www.ncbi.nlm.nih.gov/bioproject/PRJNA1503100]. All other data supporting the findings of this study are included in the article and its **Supplementary Tables**.

## References

[B1] Abu Bakar F. Ramaiya S. D. Min K. Y. (2025). Molecular insight, pathogenesis and resistance management of bacterial wilt (*Ralstonia solanacearum*) disease. Cogent Food Agric. 11, 2579885. doi: 10.1080/23311932.2025.257988537339054

[B2] Abulfaraj A. A. Mariappan K. Bigeard J. Manickam P. Blilou I. Guo X. . (2018). The Arabidopsis homolog of human G3BP1 is a key regulator of stomatal and apoplastic immunity. Life Sci. Alliance 1, e201800046. doi: 10.26508/lsa.201800046 30456348 PMC6238584

[B3] Ahmed W. Yang J. Tan Y. Munir S. Liu Q. Zhang J. . (2022). *Ralstonia solanacearum*, a deadly pathogen: Revisiting the bacterial wilt biocontrol practices in tobacco and other Solanaceae. Rhizosphere 21, 100479. doi: 10.1016/j.rhisph.2022.100479

[B4] Balamurugan A. Sakthivel K. Gautam R. Sharma S. K. Kumar A. (2021). “ *Ralstonia solanacearum*: Biology and its Management in Solanaceous Vegetable Crops,” in Rhizosphere Microbes: Soil and Plant Functions. Eds. Sharma S. K. Singh U. B. Sahu P. K. Singh H. V. Sharma P. K. ( Springer, Singapore), 259–289.

[B5] Berkey R. Bendigeri D. Xiao S. (2012). Sphingolipids and plant defense/disease: the “death” connection and beyond. Front. Plant Sci. 3, 68. doi: 10.3389/fpls.2012.00068 22639658 PMC3355615

[B6] Bolger A. M. Lohse M. Usadel B. (2014). Trimmomatic: a flexible trimmer for Illumina sequence data. Bix 30, 2114–2120. doi: 10.1093/bioinformatics/btu170 24695404 PMC4103590

[B7] Chae S. Y. Lee K. Do J. W. Hong S. C. Lee K. H. Cho M. C. . (2022). QTL mapping of resistance to bacterial wilt in pepper plants (*Capsicum annuum*) using genotyping-by-sequencing (GBS). Horticulturae 8, 115. doi: 10.3390/horticulturae802011530654563

[B8] Cho H. Song E. S. Lee Y. K. Lee S. Lee S. W. Jo A. . (2018). Analysis of genetic and pathogenic diversity of *Ralstonia solanacearum* causing potato bacterial wilt in Korea. Plant Pathol. J. 34, 23. doi: 10.5423/ppj.ft.09.2017.0203 29422785 PMC5796747

[B9] Danecek P. Bonfield J. K. Liddle J. Marshall J. Ohan V. Pollard M. O. . (2021). Twelve years of SAMtools and BCFtools. GigaScience 10, giab008. doi: 10.1093/gigascience/giab008 33590861 PMC7931819

[B10] Du H. Wen C. Zhang X. Xu X. Yang J. Chen B. . (2019). Identification of a major QTL (*qRRs-10.1*) that confers resistance to *Ralstonia solanacearum* in pepper (*Capsicum annuum*) using SLAF-BSA and QTL mapping. Int. J. Mol. Sci. 20, 5887. doi: 10.3390/plants11040538 31771239 PMC6928630

[B11] Hwang S. M. Jang K. S. Choi Y. H. Kim H. Choi G. J. (2017). Development of an efficient bioassay method to evaluate resistance of chili pepper cultivars to *Ralstonia solanacearum*. Res. Plant Dis. 23, 334–347. doi: 10.5423/rpd.2017.23.4.334

[B12] Jung Y. Han D. (2022). BWA-MEME: BWA-MEM emulated with a machine learning approach. Bix 38, 2404–2413. doi: 10.1093/bioinformatics/btac137 35253835

[B13] Kapoor S. Rana A. Devi R. Katoch A. Kumar A. (2023). Screening Bell Pepper (*Capsicum annuum* L. var. grossum Sendt.) Genotypes for Bacterial Wilt Resistance, Yield Parameters and Morphological Traits under Mid-hill. Int. J. Plant Soil Sci. 35, 389–400. doi: 10.9734/ijpss/2023/v35i23425442687827

[B14] Kwon J. S. Lee J. Shilpha J. Jang H. Kang W. H. (2024). The landscape of sequence variations between resistant and susceptible hot peppers to predict functional candidate genes against bacterial wilt disease. BMC Plant Biol. 24, 1036. doi: 10.1186/s12870-024-05742-w 39482582 PMC11529287

[B15] Kwon J. S. Nam J. Y. Yeom S. I. Kang W. H. (2021). Leaf-to-whole plant spread bioassay for pepper and *Ralstonia solanacearum* interaction determines inheritance of resistance to bacterial wilt for further breeding. Int. J. Mol. Sci. 22, 2279. doi: 10.3390/ijms22052279 33668965 PMC7956186

[B16] Lafortune D. Béramis M. Daubèze A.-M. Boissot N. Palloix A. (2005). Partial resistance of pepper to bacterial wilt is oligogenic and stable under tropical conditions. Plant Dis. 89, 501–506. doi: 10.1094/pd-89-0501 30795429

[B17] Lavale S. A. Debnath P. Mathew D. Abdelmotelb K. F. (2022). Two decades of omics in bacterial wilt resistance in Solanaceae, what we learned? Plant Stress 5, 100099. doi: 10.1016/j.stress.2022.10009942574925

[B18] Lebeau A. Daunay M. C. Frary A. Palloix A. Wang J. F. Dintinger J. . (2011). Bacterial wilt resistance in tomato, pepper, and eggplant: genetic resources respond to diverse strains in the *Ralstonia solanacearum* species complex. Phytopath 101, 154–165. doi: 10.1094/phyto-02-10-0048 20795852

[B19] Lee J. H. Lee J. Oh D. G. (2018). Resistance of pepper cultivars to *Ralstonia solanacearum* isolates from major cultivated areas of chili peppers in Korea. Hortic. Sci. Technol. 36, 569–576. doi: 10.5423/rpd.2010.16.2.141

[B20] Lee S. Chakma N. Joung S. Lee J. M. Lee J. (2022). QTL mapping for resistance to bacterial wilt caused by two isolates of *Ralstonia solanacearum* in chili pepper (*Capsicum annuum* L.). Plants 11, 1551. doi: 10.3390/plants11121551 35736702 PMC9229654

[B21] Lee J. H. Siddique M. I. Jang S. Kim G. W. Choi G. J. Kwon J. K. . (2024). Identification of QTLs associated with resistance to bacterial wilt in pepper (*Capsicum annuum* L.) through bi-parental QTL mapping and genome-wide association analysis. Sci. Hortic. 329, 112987. doi: 10.1016/j.scienta.2024.11298742574925

[B22] Li H. Handsaker B. Wysoker A. Fennell T. Ruan J. Homer N. . (2009). The sequence alignment/map format and SAMtools. Bix 25, 2078–2079. doi: 10.1093/bioinformatics/btp352 19505943 PMC2723002

[B23] Liu W. Y. Kang W. H. Kang B. C. (2013). “ Basic Information on Pepper,” in Genetics, Genomics and Breeding of Peppers and Eggplants. Eds. Kang B. C. Kole C. ( CRC Press, Boca Raton, FL), 1–15.

[B24] Naveena G. Sandeep V. Ponnam N. Kumari M. Acharya G. Srinivas P. . (2020). Genetic analysis of bacterial wilt resistance in hot pepper (*Capsicum annuum* L.). Veg. Sci. 47, 153–156. doi: 10.61180/vegsci.2020.v47.i1.30

[B25] Ro N. Haile M. Oh H. Ko H. C. Yi J. Na Y. W. . (2024). Evaluation of pepper (*Capsicum* spp.) germplasm collection for bacterial wilt (*Ralstonia solanacearum*) resistance. Agron 14, 1753. doi: 10.5423/rpd.2012.18.2.093

[B26] Safni I. Cleenwerck I. De Vos P. Fegan M. Sly L. Kappler U. (2014). Polyphasic taxonomic revision of the *Ralstonia solanacearum* species complex: proposal to emend the descriptions of *Ralstonia solanacearum* and *Ralstonia syzygii* and reclassify current *R. syzygii* strains as *Ralstonia syzygii* subsp. syzygii subsp. nov. *R. solanacearum* phylotype IV strains as *Ralstonia syzygii* subsp. indonesiensis subsp. nov. banana blood disease bacterium strains as *Ralstonia syzygii* subsp. celebesensis subsp. nov. and *R. solanacearum* phylotype I and III strains as *Ralstonia pseudosolanacearum* sp. nov. Int. J. Syst. Evol. Microbiol. 64, 3087–3103. doi: 10.1099/ijs.0.066712-0 24944341

[B27] Saucedo‐García M. Guevara‐García A. González‐Solís A. Cruz‐García F. Vázquez‐Santana S. Markham J. E. . (2011). MPK6, sphinganine and the *LCB2a* gene from serine palmitoyltransferase are required in the signaling pathway that mediates cell death induced by long chain bases in *Arabidopsis*. New Phytol. 191, 943–957. doi: 10.1111/j.1469-8137.2011.03727.x 21534970

[B28] Sharma P. Johnson M. A. Mazloom R. Allen C. Heath L. S. Lowe-Power T. M. . (2022). Meta-analysis of the *Ralstonia solanacearum* species complex (RSSC) based on comparative evolutionary genomics and reverse ecology. Microb. Genom. 8, 000791. doi: 10.1136/jnnp.64.2.227 35297758 PMC9176288

[B29] She X. Yu L. Lan G. Tang Y. He Z. (2017). Identification and genetic characterization of *Ralstonia solanacearum* species complex isolates from *Cucurbita maxima* in China. Front. Plant Sci. 8, 1794. doi: 10.3389/fpls.2017.01794 29093727 PMC5651519

[B30] Takagi H. Abe A. Yoshida K. Kosugi S. Natsume S. Mitsuoka C. . (2013). QTL‐seq: rapid mapping of quantitative trait loci in rice by whole genome resequencing of DNA from two bulked populations. Plant J. 74, 174–183. doi: 10.1111/tpj.12105 23289725

[B31] Thakur H. Sharma A. Sharma P. Rana R. (2021). An insight into the problem of bacterial wilt in *Capsicum* spp. with special reference to India. Crop Prot. 140, 105420. doi: 10.1016/j.cropro.2020.10542042574925

[B32] Tripodi P. Kumar S. (2019). “ The Capsicum crop: an introduction,” in The Capsicum Genome. Eds. Ramchiary N. Kole C. ( Springer International Publishing, Cham, Switzerland), 1–8.

[B33] Vailleau F. Genin S. (2023). *Ralstonia solanacearum*: an arsenal of virulence strategies and prospects for resistance. Annu. Rev. Phytopathol. 61, 25–47. doi: 10.1146/annurev-phyto-021622-104551 37506349

[B34] Varshney R. K. Bohra A. Yu J. Graner A. Zhang Q. Sorrells M. E. (2021). Designing future crops: genomics-assisted breeding comes of age. Trends Plant Sci. 26, 631–649. doi: 10.1016/j.tplants.2021.03.010 33893045

[B35] Zhang C. Xie W. Fu H. Chen Y. Chen H. Cai T. . (2023). Whole genome resequencing identifies candidate genes and allelic diagnostic markers for resistance to *Ralstonia solanacearum* infection in cultivated peanut (*Arachis hypogaea* L.). Front. Plant Sci. 13, 1048168. doi: 10.3389/fpls.2022.1048168 36684803 PMC9845939

